# Effect of surgical face mask wearing on tear film in women with a high body mass index

**DOI:** 10.1371/journal.pone.0277803

**Published:** 2022-11-16

**Authors:** Mana A. Alanazi, Gamal A. El-Hiti, Reem Alotaibi, Mashaaer A. Baashen, Muteb Alanazi, Raied Fagehi, Ali M. Masmali

**Affiliations:** Cornea Research Chair, Department of Optometry, College of Applied Medical Sciences, King Saud University, Riyadh, Saudi Arabia; Icahn School of Medicine at Mount Sinai, UNITED STATES

## Abstract

**Purpose:**

Due to the COVID-19 pandemic, wearing a face mask has become an essential measure to reduce the rate of virus spreading. The aim of the study was to assess the effect of wearing a surgical face mask for a short period on the tear film parameters in subjects with a high body mass index (BMI).

**Methods:**

Twenty-five females with a high BMI (31.4 ± 5.5 kg/m^2^) aged 18–35 years (22.7 ± 4.6 years) participated in the study. In addition, a control group consisting of 25 females (23.0 ± 6.7 years) with a high BMI (29.9 ± 4.1 kg/m^2^) participated in the study in which no mask was worn. The standardized patient evaluation of eye dryness (SPEED) questionnaire was completed first, followed by the phenol red thread (PRT) and tear ferning (TF) tests, before wearing the face mask. The subjects wore the face mask for 1 hour, and the measurements were performed again immediately after its removal. For the control group, the measurements were performed twice with one hour gap.

**Results:**

Significant (Wilcoxon test, p < 0.05) differences were found between the SPEED scores (p = 0.035) and the PRT measurement (p = 0.042), before and after wearing the surgical face mask. The PRT scores have improved after wearing the surgical face mask, while the dry eye symptoms detected by the SPEED questionnaire have increased. On the other hand, no significant (Wilcoxon test, p = 0.201) differences were found between the TF grades before and after wearing a surgical face mask. For the control group, no significant (Wilcoxon test, p > 0.05) differences were found between the two scores from the SPEED questionnaire and the PRT, and TF tests.

**Conclusions:**

Wearing a surgical face mask for a short duration leads to a change in volume and quality of tears as well as dry eye symptoms in women with a high BMI.

## Introduction

The tear film maintains the health of the eye and vision. Instability in the tear film functions can lead to symptoms of dry eye [1]. Dry eye is an ocular surface disorder that is more common in females and the elderly [2,3]. The prevalence of dry eye is increasing and affects a large proportion of the world population [4]. The most common causes of dry eye are insufficient tear secretion and excessive evaporation of tears [5]. A shortage in the lacrimal gland supply leads to ocular surface inflammation, abnormal tear volume, and an increase in tear osmolarity [6–8]. The dysfunction of the meibomian gland causes a shortage in lipid secretion that leads to a high tear evaporation rate (TER) [9]. Dry eye symptoms (e.g., itching, burning, photophobia, inflammation, and irradiation) are more apparent in individuals living in dry environments (e.g., high temperature and low humidity) [10,11]. The standardized patient evaluation of eye dryness (SPEED) questionnaire [12] can be used along with various tests such as phenol red thread (PRT) [13], tear break-up time (TBUT) [14], osmolarity [15], TER [16], tear ferning (TF) [17], and others to detect the symptoms of dry eye and status of the tear film. However, the diagnosis of dry eye is challenging and complex since no single test can give definite results. Therefore, various tests that assess different parameters should be used. Dry eye symptoms can be managed by increasing the eye’s comfort through various methods [18]. The most common methods involve the use of artificial tears, anti-inflammatory drugs, ocular surgery, and avoiding harsh environments. Artificial tears are the first option to treat dry eyes. They increase the viscosity of tears and lubricant and moisten the surface of the eye [19]. Vitamin A, fatty acids, and some medications help in the reduction of dry eye inflammation [20].

Body mass index (BMI) is a simple tool to detect obesity, although increases in both body fat and lean tissue can lead to a high BMI [21]. BMI is the weight (kg) of the body of an individual divided by his or her squared height (m^2^). A measurement of ≥ 25 kg/m^2^ is considered as the cut-off for a high BMI according to the World Health Organization [22]. High BMI contributes to 58% of type II diabetes, 39% of hypertension, 32% of endometrial cancer in women, 23% of ischemic stroke, 21% of ischemic heart disease, 13% of osteoarthritis, 12% of colon cancer, and 8% of postmenopausal breast cancer [22]. Moreover, a high BMI has been proven to be a risk factor for dry eye [23].

In recent years, wearing a face mask has become necessary to reduce the COVID-19 infection rate [24]. It has become a daily routine for people outdoors that has led to a huge change in our daily life habits. Therefore, the current study investigates the effect of wearing a surgical face mask for a short duration on the tear film in subjects with a high BMI.

## Materials and methods

### Study design, subjects, and ethics

This observational nonrandomized comparative study was carried out in the Clinics of the College of Applied Medical Sciences, Riyadh. The measurements took place indoors in which the temperature was fixed at 20°C and the humidity was around 15%. The size of the sample has been calculated as 24 subjects. The probability to detect a relationship between the independent and the dependent variables at a two-sided 0.05 significance level was 80%. Twenty-five females with a high BMI (31.4 ± 5.5 kg/m^2^) aged 18–35 years (22.7 ± 4.6 years) were recruited. In addition, a control group consisting of 25 females (23.0 ± 6.7 years) with a high BMI (29.9 ± 4.1 kg/m^2^) participated in the study in which no mask was worn. Subjects with a history of ocular surgery, contact lens wearers, smokers, and pregnant or breastfeeding women were excluded. In addition, the exclusion criteria included subjects with thyroid gland dysfunction, a high blood cholesterol level (> 4 mmol/L), high refractive errors (more than ± 2.00 D), vitamin A and D deficiencies, hypertension, anemia, or diabetes. The tests were performed by the same examiner at the Clinics of the College of Applied Medical Sciences, Riyadh. The SPEED questionnaire was completed first, followed by the PRT and TF tests, before wearing the surgical face mask. The Band Med three layers surgical face mask (50 pack, China) with an invisible metal strip that allows the nose to fit snugly and comfortably breathing was used. The subjects wore the face mask for 1 hour, and the measurements were performed again immediately after its removal. The masks covered the nose, and all subjects followed the protocol. For the control group, the measurements were performed twice with one hour gap for comparison. During the one-hour session, the subjects carried out no activities. The tests were carried out on the right eye of each subject, and a 5-minute gap was allowed between the tests. It is not likely that the material of the surgical face mask would induce face, skin, and eye irritation, however, such a possibility has not been tested. The current research is a preliminary study to assess the effect of wearing a face mask on the tear film in subjects with a high BMI. The study was conducted in accordance with the Declaration of Helsinki and approved by King Saud University Ethics Committee (E-21-6471). Informed written consent was obtained from all participants.

### SPEED questionnaire

The SPEED questionnaire was completed first by all subjects. It contains 12 questions in three different sections (symptoms, frequency, and severity). It is used as a validated tool for the detection of dry eye symptoms [25]. The score of the SPEED questionnaire is calculated from the frequency and severity sections [26]. The total SPEED score is obtained from the summation of the responses in the sections of frequency (from 0 to 3) and severity (from 0 to 4) divided by 28 [26]. Dry eye was defined for a SPEED questionnaire score more than 4.

### PRT test

The PRT test was performed using a cotton thread (Zone-Quick, Showa Yakuhin Kako Co, Ltd., Tokyo, Japan) containing a pH indicator. The cotton thread with a 3mm bent end was gently inserted in the lower fornix for 15 seconds. The thread changes color when it comes in contact with tears. The red-colored portion of the thread was measured, and dry eye was defined as measurements less than 10 mm [27].

### TF test

A glass capillary tube (10 μL; Merck, Schnelldorf, Germany) was used to collect a tear sample (1 μL) from the lower meniscus of the right eye. The tears collected were dried in a controlled environment (23°C and with a humidity of 10%) for 10 minutes. A digital microscope (Olympus DP72; Tokyo, Japan) was used to observe and capture the TF patterns at a magnification of 10×. The TF patterns for each tear sample were graded based on the five-point TF grading scale (from 0 to 4), using increments of 0.1 [28–32]. Dry eye was defined for a TF grade ≥ 2. The TF grading was done by two independent researchers in which the second one was masked. The TF scores from the two researchers were similar. The TF grades were averaged and recorded to one decimal place.

### Statistical analysis

Microsoft Excel 2016 (Microsoft Office, Microsoft Corp., Redmond, WA, USA) was used to collect the data. The Statistical Package for the Social Sciences software (IBM Software, version 22, Armonk, NY, USA) was used to analyze the data [33]. The independent samples t-test has been used to compare the measurements of tear film parameters. The data has been identified as statistically significant when p < 0.05. The data were abnormally distributed (Shapiro-Wilk test; p < 0.05). Therefore, the median (interquartile range; IQR) was used to represent the averages.

## Results

The median (IQR) for the SPEED, PRT, and TF scores for the subjects with a high BMI (pre and post-wearing the face mask) are shown in Table 1 and those for the control group (with an hour gap) are shown in Table 2.

**Table 1:** The median (IQR) for the SPEED, PRT, and TF scores in subjects with a high BMI (n = 25).

Parameter	Pre-wearing the mask	Post-wearing the mask	p value
**SPEED** *	5.0 (7.5)	3.1 (7.5)	0.035
**PRT (mm)** *	23.0 (12.5)	27.6 (9.5)	0.042
**TF**	1.7 (0.8)	1.6 (0.9)	0.201

* Significant difference (Wilcoxon test).

**Table 2:** The median (IQR) for the SPEED, PRT, and TF scores in the control group (n = 25).

Parameter	Measurement 1	Measurement 2	p value*
**SPEED**	5.3 (2.3)	5.4 (2.5)	0.268
**PRT (mm)**	22.5 (6.5)	22.7 (4.6)	0.321
**TF**	1.8 (0.5)	1.8 (0.6)	0.521

* Wilcoxon test.

The PRT scores have improved after wearing the surgical face mask, while the dry eye symptoms detected by the SPEED questionnaire have increased. Significant (Wilcoxon test, p < 0.05) differences were found between the SPEED scores (p = 0.035) and the PRT measurements (p = 0.042) in subjects with a high BMI before and after wearing a face mask. On the other hand, no significant (Wilcoxon test, p = 0.201) difference was found between the TF grades before and after wearing a surgical face mask. For the control group, no significant (Wilcoxon test, p > 0.05) differences were found between the two scores from the SPEED questionnaire and the PRT, and TF tests.

The SPEED scores recorded after wearing the mask had increased in 6 subjects (24%), decreased in 10 (40%), and were unchanged in 9 cases (36%). For the PRT, the score increased in 13 subjects (52%), decreased in 8 subjects (32%), and was unchanged in four cases after wearing the mask. For the TF test, the grade after wearing the mask increased in the majority of subjects (n = 14; 56%) and decreased in 11 cases (44%). The TF test indicated that the quality of tears was reduced in the majority of subjects in the study group as a result of wearing the mask. Clearly, the correlations between the SPEED, TF, and PRT scores are weak since each one of them assesses a different parameter. For the control group, no change in the quality or volume of tears was observed.

**Fig 1:**
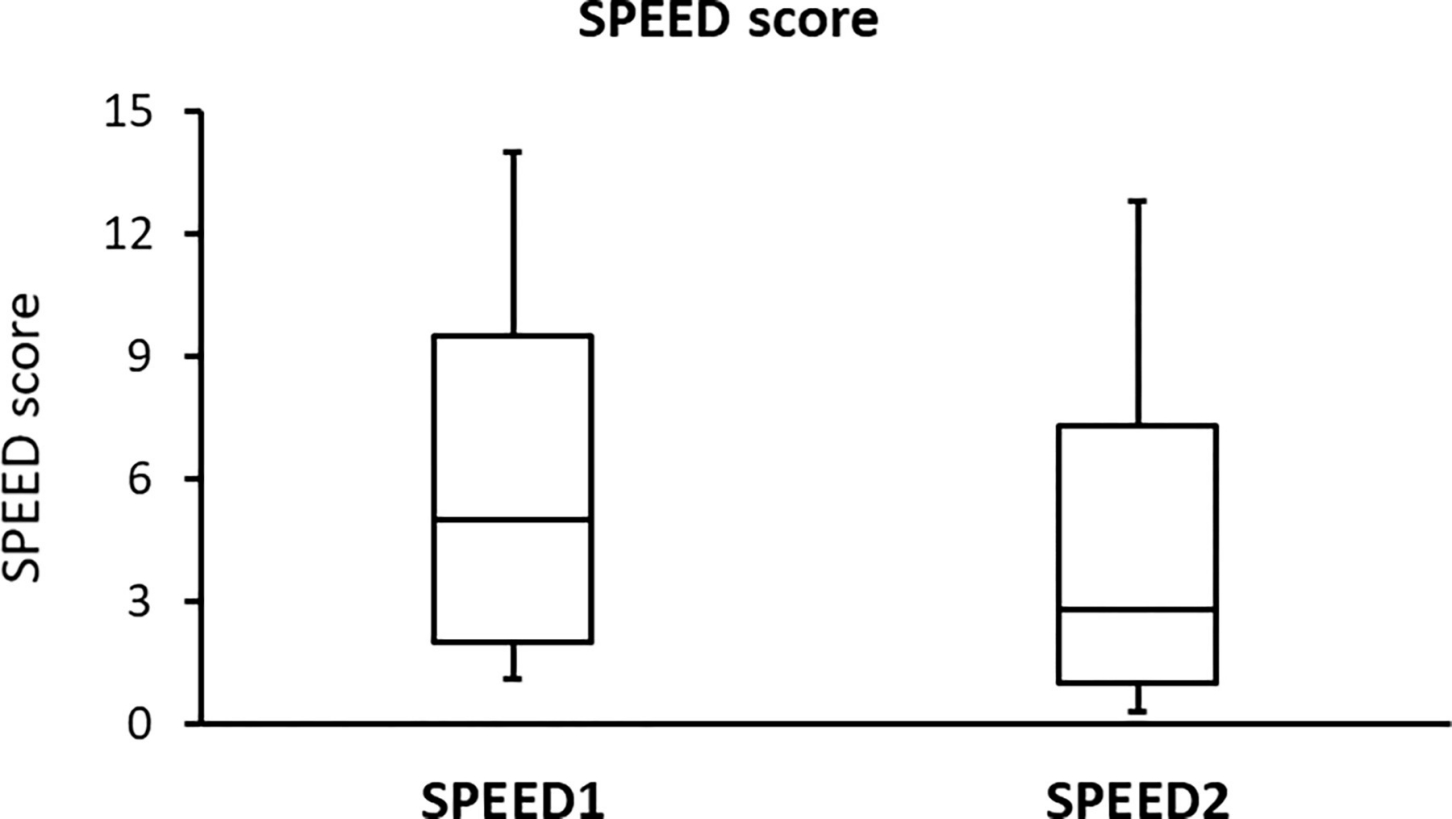
Side-by-side boxplots for the SPEED scores for the subjects with a high BMI before (SPEED1) and after (SPEED2) wearing a surgical face mask.

**Fig 2:**
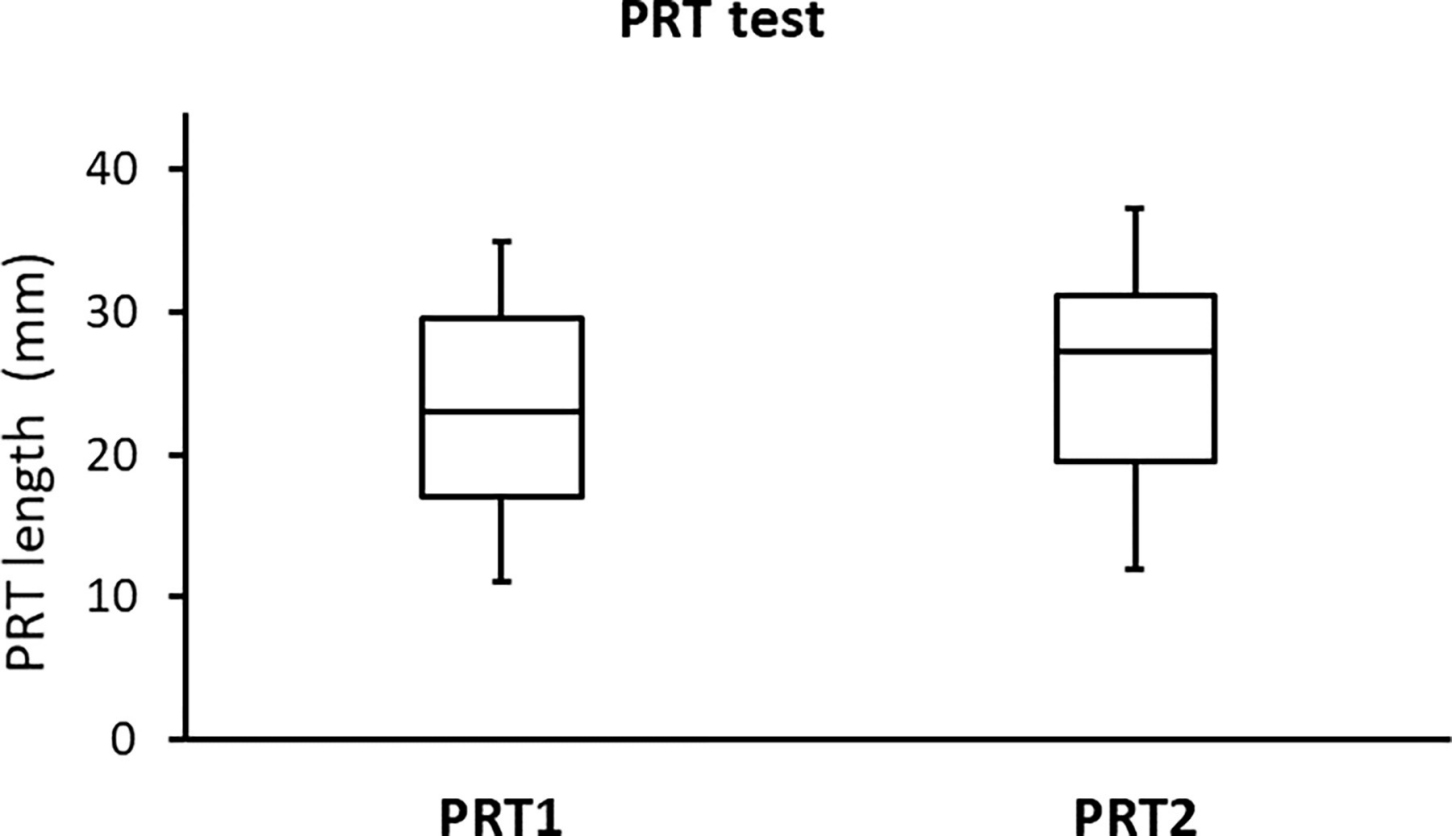
Side-by-side boxplots for the PRT scores for the subjects with a high BMI before (PRT1) and after (PRT2) wearing a surgical face mask.

**Fig 3:**
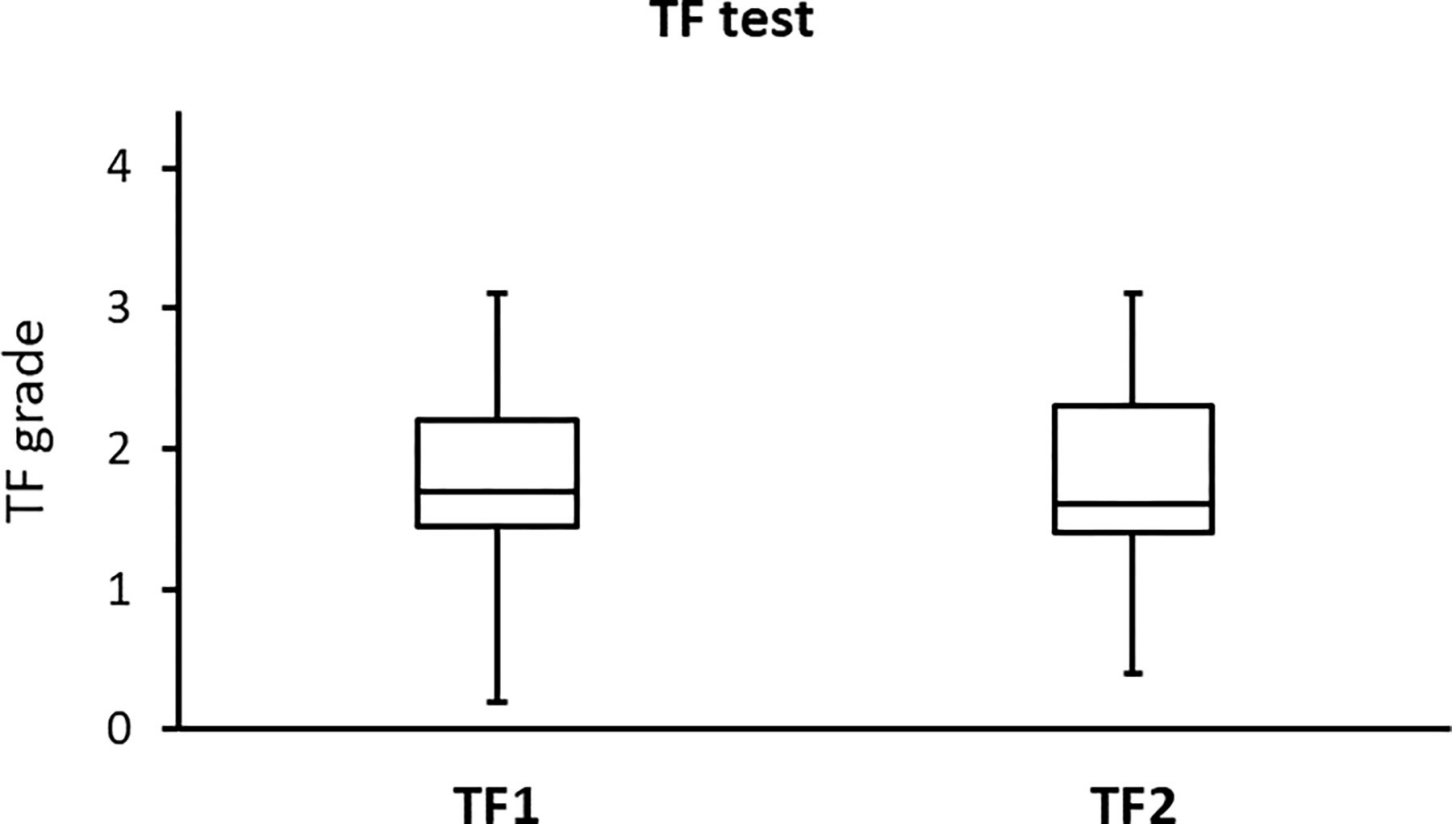
Side-by-side boxplots for the TF grades for the subjects with a high BMI before (TF1) and after (TF2) wearing a surgical face mask.

**Fig 4:**
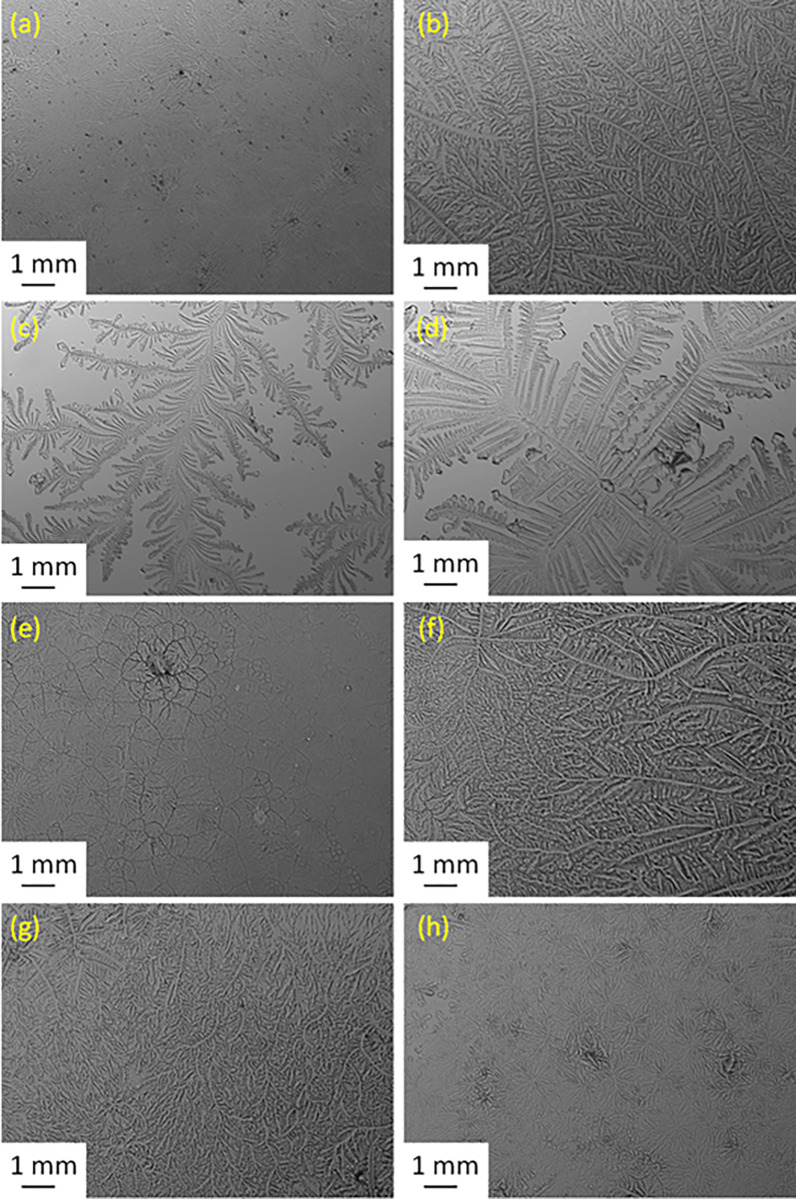
Representative TF images of the tears collected from four subjects with a high BMI before (a, c, e, and g) and after (b, d, f, and h) wearing a surgical face mask.

The side-by-side boxplots for the SPEED, PRT, and TF scores in subjects with a high BMI (before and after wearing a face mask) are shown in Figs 1–3, respectively. Representative TF patterns of the tears collected from four subjects before (a, c, e, and g, respectively) and after (b, d, f, and h, respectively) wearing a surgical face mask are shown in Fig 4.

## Discussion

The current study suggests an association between wearing a medical face mask and tear film parameters. Dry eye symptoms increased significantly after wearing a face mask even for a short duration (1 hour). On the other hand, the tear volume increased significantly as a result of wearing a face mask. Dry eye has been associated with a high BMI [23,34–37]. For example, a study conducted in 20 subjects with a high BMI (31.8 (5.2) kg/m^2^) showed a significant (p < 0.05) decrease in NITBUT scores and a significant increase in TF grades compared with the control group [23]. No significant (p > 0.05) differences were seen between the study and control groups for the ocular surface disease index (OSDI), PRT, and THM scores. In addition, a study conducted among 305 subjects using a short dry eye questionnaire suggested a medium correlation (r = 0.34, p = 0.003) between dry eye and body fat percentage [38]. However, a recent study conducted in a very large Japanese population (n = 85,264) that included males and females (40–74 years) suggested an inverse relationship between the prevalence of dry eye and a high BMI [39].

A recent study suggested a relationship between wearing a mask and ocular tear film instability [40]. The study was conducted among individuals wearing a mask in a medical practice in which the OSDI was used to assess dry eye symptoms. Ocular discomfort was found to be associated with wearing face masks. The severity of the symptoms was highly dependent on the type of mask used and the duration of its wearing [41]. The majority of subjects suffer from dry eye discomfort, such as burning, tearing, itching, blurred vision, and redness. The symptoms were more severe in subjects who wore regular protective medical masks compared to non-medical ones. In addition, the majority of subjects showed skin irritation, shortness of breath, physical disturbance, and headache [41]. Another cross-sectional study that was conducted with 31 subjects, mainly females (n = 30), showed a decrease in tear film stability after wearing face masks. The average NITBUT score was 7.8 ± 5.6 s before wearing a mask and decreased significantly (p = 0.006) to 6.2 ± 6.8 s afterwards [42].

Very recently, it has been reported that wearing an N95 surgical face mask leads to a negative effect on the scores collected from Schirmer and TBUT tests. The tear film parameters worsen as the duration of wearing the mask increases [43]. Wearing the face mask for a long duration (e.g., more than 8 h) leads to an increase in dry eye symptoms that were detected using the OSDI a significant (p = 0.01) decrease in the TBUT [44]. Clearly, wearing a face mask for long duration has a negative effect on the tear film and daily life activities [45–47]. People infected with COVID-19 have signs of dry eye and blepharitis [48,49].

The air blowing upward from the mask into the eye might cause of dry eye symptoms. The air blowing accelerates tear evaporation, hyperosmolarity, and tear film disturbance. As a result, symptoms of ocular surface irritation and inflammation have been felt by individuals wearing masks [40–42]. The thickness of the lipid layer determines the severity of dry eye symptoms [50]. Indeed, the prevalence of dry eye was found to be high in individuals living in harsh environments due to high airflow [51,52]. Therefore, dry eye symptoms should be managed in subjects wearing face masks for a long duration. In addition, measures should be taken to prevent any ocular damage in subjects with severely dry eyes as a result of mask-wearing.

### Study limitations

The limitations of the current study include the use of a low number of subjects, the inclusion of only females from Riyadh, the short duration of mask-wearing (1 hour), and the testing of only a single type of mask (surgical). Therefore, a future study is still needed to test the effect of gender and different types of masks (e.g., surgical and fabric), and longer duration on the tear film parameters using various objective and subjective tests.

## Conclusions

Wearing a surgical face mask for a short duration leads to a change in in volume and quality of tears as well as dry eye symptoms in women with a high body mass index.

## References

[1] Bron AJ, Tomlinson A, Foulks GN, JPepose JS, Baudouin C, Geerling G, et al. Rethinking dry eye disease: a perspective on clinical implications. *Ocul Surf* 2014; 12: S1–S31. doi: 10.1016/j.jtos.2014.02.002 . 24725379

[2] Sharma A, Hindman HB. Aging: a predisposition to dry eyes. *J Ophthalmol* 2014; 2014: 781683. doi: 10.1155/2014/781683 . 25197560 PMC4150485

[3] de Paiva CS. Effects of aging in dry eye. *Int Ophthalmol Clin* 2017; 57: 47–64. doi: 10.1097/IIO.0000000000000170 . 28282314 PMC5347479

[4] Lemp MA. Advances in understanding and managing dry eye disease. *Am J Ophthalmol* 2008; 146: 350–356. doi: 10.1016/j.ajo.2008.05.016 . 18599017

[5] Phadatare SP, Momin M, Nighojkar P, Askarkar S, Singh KK. A comprehensive review on dry eye disease: diagnosis, medical management, recent developments, and future challenges. *Adv Pharm* 2015; 2015: 704946. 10.1155/2015/704946.

[6] Stern ME, Gao J, Siemasko KF, Beuerman RW, Pflugfelder SC. The role of the lacrimal functional unit in the pathophysiology of dry eye. *Exp Eye Res* 2004; 78: 409–416. doi: 10.1016/j.exer.2003.09.003 . 15106920

[7] Niederkorn JY, Kaplan HJ. Immune response and the eye. *Chem Immunol Allergy* 2007; 92: 176–184. 10.1159/isbn.978-3-318-01404-4. 17264477

[8] Johnson ME, Murphy PJ. Changes in the tear film and ocular surface from dry eye syndrome. *Prog Retin Eye Res* 2004; 23: 449–474. doi: 10.1016/j.preteyeres.2004.04.003 . 15219877

[9] Foulks GN, Nichols KK, Bron AJ, Holland EJ, McDonald MB, Nelson JD. Improving awareness, identification, and management of meibomian gland dysfunction. *Ophthalmology* 2012; 119: S1–S12. doi: 10.1016/j.ophtha.2012.06.064 . 23034341

[10] Ohashi Y, Ishida R, Kojima T, Goto E, Matsumoto Y et al. Abnormal protein profiles in tears with dry eye syndrome. *Am J Ophthalmol* 2003; 136: 291–299. doi: 10.1016/s0002-9394(03)00203-4 . 12888052

[11] Kaercher T, Bron A. Classification and diagnosis of dry eye. In: Surgery for the Dry Eye. Geerling G, Brewitt H, Eds; Developments in Ophthalmology 2008; 41: 36–53. 10.1159/000131069. 18453760

[12] Finis D, Pischel N, König C, Hayajneh J, Borrelli M, Schrader S, et al. Comparison of the OSDI and SPEED questionnaires for the evaluation of dry eye disease in clinical routine. *Ophthalmologe* 2014; 111: 1050–1056. doi: 10.1007/s00347-014-3042-z . 25030896

[13] Tomlinson A, Blades KJ, Pearce EI. What does the phenol red thread test actually measure? *Optom Vis Sci* 2001; 78: 142–146. doi: 10.1097/00006324-200103000-00005 . 11327674

[14] Martin E, Oliver KM, Pearce EI, Tomlinson A, Simmons P, Hagan S. Effect of tear supplements on signs, symptoms and inflammatory markers in dry eye. *Cytokine* 2018; 105: 37–44. doi: 10.1016/j.cyto.2018.02.009 . 29452970

[15] Fagehi R, Al-Bishry AB, Alanazi MA, Abusharha A, El-Hiti GA, Masmali AM. Investigation of the repeatability of tear osmolarity using an I-PEN osmolarity device. *Taiwan J Ophthalmol* 2021; 11: 168–174. doi: 10.4103/tjo.tjo_65_20 . 34295623 PMC8259515

[16] Fagehi R, El-Hiti GA, Alsubaie MH, Abusharha A, Alanazi MA, Masmali AM, et al. Measurements of tear evaporation rate in subjects with refractive errors using a portable evaporimeter. *Healthcare* 2022; 10: 405. doi: 10.3390/healthcare10020405 . 35207019 PMC8872598

[17] Masmali AM, Alanazi SA, Alotaibi AG, Fagehi R, Abusharaha A, El-Hiti GA. The acute effect of a single dose of green tea on the quality and quantity of tears in normal eye subjects. *Clin Ophthalmol* 2019; 13: 605–610. doi: 10.2147/OPTH.S201127 . 31040641 PMC6462167

[18] Methodologies to diagnose and monitor dry eye disease: report of the Diagnostic Methodology Subcommittee of the Inter-national Dry Eye Workshop (2007). *The *Ocul Surf* 2007*; 5: 108–152. doi: 10.1016/s1542-0124(12)70083-6 . 17508118

[19] Pucker AD, Ng SM, Nichols JJ. Over the counter (OTC) artificial tear drops for dry eye syndrome. **Cochrane Database Syst*. *Rev**. 2016; 2: CD009729. doi: 10.1002/14651858.CD009729.pub2 . 26905373 PMC5045033

[20] Perry HD, Doshi-Carnevale S, Donnenfeld ED, Solomon R, Biser SA, Bloom AH. Efficacy of commercially available topical cyclosporine A 0.05% in the treatment of meibomian gland dysfunction. *Cornea* 2006; 25: 171–175. doi: 10.1097/01.ico.0000176611.88579.0a . 16371776

[21] James WPT, Jackson-Leach R, Mhurchu CN, Kalamara E, Shayeghi M, Rigby NJ, et al. Over-weight and obesity (high body mass index). Comparative quantification of health risks: global and regional burden of disease attributable to selected major risk factors. In Comparative Quantification of Health Risks Global and Regional Burden of Disease Attributable to Selected Major Risk Factors, Ezzati M, Lopez AD Rodgers, Murry CJL, Eds; World Health Organization: Geneva, Switzerland, 2004; Volume 1, Chapter 8, pp. 497–596.

[22] Appropriate body-mass index for Asian populations and its implications for policy and intervention strategies. *Lancet* 2004; 363: 157–163. doi: 10.1016/S0140-6736(03)15268-3 . 14726171

[23] Alanazi SA. Assessment of tear film in subjects with a high body mass index. *Clin Optom* 2019; 11: 77–84. doi: 10.2147/OPTO.S218109 . 31440111 PMC6666370

[24] Brooks JT, Butler JC. Effectiveness of mask wearing to control community spread of SARS-CoV-2. *JAMA* 2021; 325: 998–999. doi: 10.1001/jama.2021.1505 . 33566056 PMC8892938

[25] Asiedu K. Rasch analysis of the standard patient evaluation of eye dryness questionnaire. *Eye Contact Lens* 2017; 43: 394–398. doi: 10.1097/ICL.0000000000000288 . 27341091

[26] Ngo W, Situ P, Keir N, Korb D, Blackie C, Simpson T. Psychometric properties and validation of the standard patient evaluation of eye dryness questionnaire. *Cornea* 2013; 32: 1204–1210. doi: 10.1097/ICO.0b013e318294b0c0 . 23846405

[27] Masmali A, Alqahtani TA, Alharbi A, El-Hiti GA. Comparative study of repeatability of phenol red thread test versus Schirmer’s test in normal adults in Saudi Arabia. *Eye Contact Lens* 2014; 40: 127–131. doi: 10.1097/ICL.0000000000000025 . 24681611

[28] Alanazi MA, El-Hiti GA, Al-Madani A, Fagehi R. Analysis of tear ferning patterns in young female subjects with refractive errors. *J. Ophthalmol* 2021; 2021: 9524143. doi: 10.1155/2021/9524143 33552597 PMC7847330

[29] Masmali AM, Maeni YA, El-Hiti GA, Murphy PJ, Almubrad T. Investigation of ocular tear ferning in controlled and uncontrolled diabetic subjects. *Eye Contact Lens* 2018; 44: S70–S75. doi: 10.1097/ICL.0000000000000419 . 28945647

[30] Masmali AM, Al-Shehri A, Alanazi SA, Abusharaha A, Fagehi R, El-Hiti GA. Assessment of tear film quality among smokers using tear ferning patterns. *J Ophthalmol* 2016; 2016: 8154315. doi: 10.1155/2016/8154315 . 28003910 PMC5143709

[31] Masmali AM, Al-Qhtani S, Al-Gasham TM, El-Hiti GA, Purslow C, Murphy PJ. Application of a new grading scale for tear ferning in non-dry eye and dry eye subjects. *Cont Lens Anterior Eye* 2015; 38: 39–43. doi: 10.1016/j.clae.2014.09.007 . 25305710

[32] Masmali A, Al-Bahlal JM, El-Hiti GA, Akhtar S, Purslow C, Murphy PJ, et al. Repeatability and diurnal variation of tear ferning test. Eye Contact Lens 2015; 41: 262–267. doi: 10.1097/ICL.0000000000000116 . 25603440

[33] Cohen J. *Statistical Power Analysis for the Behavioral Sciences*; 2nd ed.; Lawrence Erlbaum Associates: Hillsdale, NJ, USA, 1988.

[34] Yang W-J, Yang Y-N, Cao J, Man Z-H, Yuan J, Xiao X, et al. Risk factors for dry eye syndrome: a retrospective case-control study. *Optom Vis Sci* 2015; 92: e199–205. doi: 10.1097/OPX.0000000000000541 . 25756335

[35] Ahn JM, Lee SH, Rim THT, Park RJ, Yang HS, Kim TI, et al. Epidemiologic Survey Committee of the Korean Ophthalmological Society. Prevalence of and risk factors associated with dry eye: the Korea National Health and Nutrition Examination Survey 2010–2011. *Am J Ophthalmol* 2014; 158: 1205–1214. doi: 10.1016/j.ajo.2014.08.021 . 25149910

[36] Malet F, Le Goff M, Colin J, Schweitzer C, Delyfer M-N, Korobelnik J-F, et al. Dry eye disease in French elderly subjects: the Alienor Study. *Acta Ophthalmol* 2014; 92: e429–436. doi: 10.1111/aos.12174 . 23742664

[37] Uchino M, Nishiwaki Y, Michikawa T, Shirakawa K, Kuwahara E, Yamada M, et al. Prevalence and risk factors of dry eye disease in Japan: Koumi study. *Ophthalmology* 2011; 118: 2361–2367. doi: 10.1016/j.ophtha.2011.05.029 . 21889799

[38] Ho KC, Jalbert I, Watt K, Golebiowski B. A possible association between dry eye symptoms and body fat: a prospective, cross-sectional preliminary study. *Eye Contact Lens* 2017; 43: 245–252. doi: 10.1097/ICL.0000000000000275 . 27243357

[39] Yamanishi R, Sawada N, Hanyuda A, Uchino M, Kawashima M, Yuki K, et al. Relation between body mass index and dry eye disease: The Japan Public Health Center-Based prospective study for the next generation. *Eye Contact Lens* 2021; 47: 449–455. doi: 10.1097/ICL.0000000000000814 . 34310488

[40] Moshirfar M, West BW, Marx DP. Face mask-associated ocular irritation and dryness. *Ophthalmol Ther* 2020; 9: 397–400. doi: 10.1007/s40123-020-00282-6 . 32671665 PMC7362770

[41] Marinova E, Dabov D, Zdravkov Y. Ophthalmic complaints in face-mask wearing: prevalence, treatment, and prevention with a potential protective effect against SARS-CoV-2. *Biotechnol Biotechnol Equip* 2020; 34: 1323–1335. 10.1080/13102818.2020.1838323.

[42] Arriola-Villalobos P, Burgos-Blasco B, Vidal-Villegas B, Oribio-Quinto C, Ariño-Gutiérrez M, Diaz-Valle D, et al. Effect of face mask on tear film stability in eyes with moderate-to-severe dry eye disease. *Cornea* 2021; 40: 1336–1339. doi: 10.1097/ICO.0000000000002734 . 34481410

[43] Shalaby HS, Eldesouky MEE. Effect of facemasks on the tear film during the COVID-19 pandemic. *Eur J Ophthalmol* 2022; ahead of print. doi: 10.1177/11206721221110010 . 35733391 PMC9289170

[44] Esen Baris M, Guven Yilmaz S, Palamar M. Impact of prolonged face mask wearing on tear break-up time and dry eye symptoms in health care professionals. *Int Ophthalmol* 2022; 42: 2141–2144. doi: 10.1007/s10792-022-02213-9 . 35119609 PMC8815392

[45] Wu P, Duan F, Luo C, Lui Q, Qu X, Liang L, et al. Characteristics of ocular findings of patients with coronavirus disease 2019 (COVID-19) in Hubei Province, China. *JAMA Ophthalmol* 2020; 138: 575–578. doi: 10.1001/jamaophthalmol.2020.1291 . 32232433 PMC7110919

[46] Sun C, Wang Y, Liu G, Liu Z. Role of the eye in transmitting human coronavirus: what we know and what we do not know. *Front in Public Health* 2020; 8: 155. doi: 10.3389/fpubh.2020.00155 . 32391309 PMC7193031

[47] Freni F, Meduri A, Gazia F, Nicastro V, Galletti C, Aragona P, et al. Symptomatology in head and neck district in coronavirus disease (COVID-19): a possible neuroinvasive action of SARS-CoV-2. *Am J Otolaryngol* 2020; 41: 102612. doi: 10.1016/j.amjoto.2020.102612 . 32574896 PMC7301823

[48] dell’Omo R., Filippelli M., Virgili G., Bandello F, Querques G, Lanzetta P, et al. Effect of COVID-19-related lockdown on ophthalmic practice in Italy: A report from 39 institutional centers. *Eur J Ophthalmol* 2022; 32: 695–703. doi: 10.1177/11206721211002442 . 33724078

[49] Meduri A, Oliverio GW, Mancuso G, Giuffrida A, Guarneri C, Rullo EV, et al. Ocular surface manifestation of COVID-19 and tear film analysis. *Sci Rep* 2020; 10: 20178. doi: 10.1038/s41598-020-77194-9 . 33214658 PMC7677531

[50] Foulks GN. The correlation between the tear film lipid layer and dry eye disease. *Surv Ophthalmol* 2007; 52: 369–374. doi: 10.1016/j.survophthal.2007.04.009 . 17574063

[51] Moss SE, Klein R, Klein BEK. Prevalence of and risk factors for dry eye syndrome. *Arch Ophthalmol* 2000; 118: 1264–1268. doi: 10.1001/archopht.118.9.1264 . 10980773

[52] Wolkoff P, Nøjgaard JK, Franck C, Skov P. The modern office environment desiccates the eyes? *Indoor Air* 2006; 16: 258–265. doi: 10.1111/j.1600-0668.2006.00429.x . 16842606

