# Supplementary material for: Effect of surgical face mask wearing on tear film in women with a high body mass index
Source: PLoS One. 2022 Nov 16;17(11):e0277803. doi: 10.1371/journal.pone.0277803 (PMC9668155; doi:10.1371/journal.pone.0277803)
Supplement: Attachment — Submitted filename: Review article surgical masks high BMI and dry eye.pdf [file pone.0277803.s001.pdf]

The full title should be Effect of surgical face mask wearing on tear film in **women** with a high body mass index

Methods: high BMI (29.9 ± 4.1 years)- Should not state **years but kg/m 2 instead.**

What were the subjects doing in the hour between the tests? Are eyes open/closed? Same activities? Who tested the subjects? Same person? How many people analyzed the results?

Intro: They increase the viscosity of tears but result in blurry vision for a short duration [19] – This is a problematic phrasing. Furthermore, the reference does not fit. The latter involves examining two different drops on Rabbits.
A measurement of □ 25
kg/m2 is considered the cut-off for a high BMI according to the **WHO** [22].
Abbreviation not specified.

In recent years, wearing a face mask has become necessary to reduce the COVID-19 infection rates. Missing reference.

Methods: No specification on the way participants wore the mask – above or below the nose and who were excluded if they broke protocol.

The TF patterns for each tear sample were graded
based on the five-point TF grading scale – Was this done by a single observer or a few? This is important due to the test's nonlinear and semi-qualitative analysis.

There is no justification for the suggested number of participants in the methods section.

Results: Dry eye symptoms increased according to speed, and PRT improved. This needs further discussion. TF did not change. We do not expect the lacrimal gland to alter. How is this explained?

Discussion:

The authors suggest that parameters in the tear film changed but did not offer a hypothesis for their findings.

Conclusion –

Missing data regarding osmolarity, electrolytes, and the parameters that the writers imply changed.

In the **literature review**, important articles such as:

Shalaby HS, Eldesouky MEE. Effect of facemasks on the tear film during the COVID-19 pandemic. Eur J Ophthalmol. 2022 Jun 22:11206721221110010. Doi: 10.1177/11206721221110010. Epub ahead of print. PMID: 35733391; PMCID: PMC9289170.

And

Esen Baris M, Guven Yilmaz S, Palamar M. Impact of prolonged face mask wearing on tear break-up time and dry eye symptoms in health care professionals. Int Ophthalmol. 2022 Jul;42(7):2141-2144. DOI: 10.1007/s10792-022-02213-9. Epub 2022 Feb 4. PMID: 35119609; PMCID: PMC8815392

Are missing.
