# Supplementary material for: Effect of surgical face mask wearing on tear film in women with a high body mass index
Source: PLoS One. 2022 Nov 16;17(11):e0277803. doi: 10.1371/journal.pone.0277803 (PMC9668155; doi:10.1371/journal.pone.0277803)
Supplement: Attachment — Submitted filename: Effect of surgical face mask wearing on tear film in individuals with a high body mass index.docx [file pone.0277803.s002.docx]

**The authors presented an interesting study evaluating the effect of wearing a surgical face mask for a short period of time on the tear film parameters in subjects with a high body mass index. The manuscript is clear, its topic is original in content, and the conclusions are consistent with the evidence presented. The manuscript is with merit and the findings are worth reporting, but the authors should address the following comments before publication**

**Introduction:**

- Line 58: “tear break-up time (TBUT) [14], osmolarity [15], TER [16], tear ferning (TF) [17]” **Authors need to explain the abbreviation “TER”.**

**Methods:**

- Line 82: **“**Methods and methods”- please correct this **repetition**.
- Lines 85-86: “in a controlled environment of temperature and humidity” **Authors need to explain what kind of environment was it- what temperature, what humidity, was it indoor or outdoor, were patients remaining indoor for 1 hour?**
- Line 92: “high refractive errors” **– the authors should expand this paragraph by providing additional details about what they mean by high refractive error?**
- Line 93-94: “The medical record of each subject was checked including blood analysis.”- **Authors are encouraged to** **specify if you checked blood work that was already done, were they looking for anything specific? Did they order any blood work if there wasn’t any results available in subjects’ charts?**

**Results:**

- Lines 158-159: “For the control group, no significant differences were found between the two scores from the SPEED questionnaire and the PRT, and TF tests.” – **Authors need to show SPEED, PRT, and TF scores for the control group.**
- Line 165-166: “The TF test indicated that the quality of tears was reduced in the majority of subjects in the study group as a result of wearing the mask.” – **What about control group again? Authors need to provide more details about test results in control group.**

**Discussion:**

- Lines 192-194: “No significant differences were seen between the study and control groups for the SODI, PRT, and THM scores.” **Please explain abbreviations SODI, THM as they were not used in manuscript before.**
- **The authors should provide discussion on the limitations of their study. For example: is there any significance in gender differences in similar studies in the literature?**
