# Supplementary material for: Effect of surgical face mask wearing on tear film in women with a high body mass index
Source: PLoS One. 2022 Nov 16;17(11):e0277803. doi: 10.1371/journal.pone.0277803 (PMC9668155; doi:10.1371/journal.pone.0277803)
Supplement: Attachment — Submitted filename: Second review.docx [file pone.0277803.s003.docx]

The author did correct most of the suggestions. This article looks better.

Nevertheless, some corrections are still not self-explanatory.

For example, even after the reviewer asked for a justification for the sample size, the author changed the manuscript to: "The size of the sample has been calculated as 25 subjects".

There is no power analysis. There is no mention of the software used and this is not reproducible.

Good luck.
