# Supplementary material for: Effect of surgical face mask wearing on tear film in women with a high body mass index
Source: PLoS One. 2022 Nov 16;17(11):e0277803. doi: 10.1371/journal.pone.0277803 (PMC9668155; doi:10.1371/journal.pone.0277803)
Supplement: Attachment — Submitted filename: Final decision .docx [file pone.0277803.s004.docx]

The manuscript looks much better, including statistical analysis and an explanation of the methods.

There are no further comments.
